# Knowledge discovery from patients’ behavior via clustering-classification algorithms based on weighted eRFM and CLV model: An empirical study in public health care services

**Published:** 2016

**Authors:** Zeinab Zare Hosseini, Mahdi Mohammadzadeh

**Affiliations:** a Department of Engineering & Technology, Payame Noor University, Tehran, IRAN.; b Shahid Beheshti university of medical sciences, Faculty of pharmacy, Tehran, IRAN.

**Keywords:** Hospital, Knowledge discovery, CRM, Data mining, RFM, Patient behavior

## Abstract

The rapid growing of information technology (IT) motivates and makes competitive advantages in health care industry. Nowadays, many hospitals try to build a successful customer relationship management (CRM) to recognize target and potential patients, increase patient loyalty and satisfaction and finally maximize their profitability. Many hospitals have large data warehouses containing customer demographic and transactions information. Data mining techniques can be used to analyze this data and discover hidden knowledge of customers. This research develops an extended RFM model, namely RFML (added parameter: Length) based on health care services for a public sector hospital in Iran with the idea that there is contrast between patient and customer loyalty, to estimate customer life time value (CLV) for each patient. We used Two-step and K-means algorithms as clustering methods and Decision tree (CHAID) as classification technique to segment the patients to find out target, potential and loyal customers in order to implement strengthen CRM. Two approaches are used for classification: first, the result of clustering is considered as Decision attribute in classification process and second, the result of segmentation based on CLV value of patients (estimated by RFML) is considered as Decision attribute. Finally the results of CHAID algorithm show the significant hidden rules and identify existing patterns of hospital consumers.

## Introduction

Customer relationship management (CRM) denotes that managerial efforts to business processes and technologies that designed to understand the customers of a firm (1). Recently, the rapid changes in IT have been accompanied by the issue of CRM, as well as data mining techniques within various industries, which have become the focal point of challenges in marketing strategy and implementation (2). The effective and efficient utilization of IT is a method for implementing a successful CRM strategy to understand customer needs, satisfy customers demands, mine for valuable customer information, identify target and potential customers, realize the maximum customer value, increase customer loyalty, and finally maximize profits (3). Precise evaluation of customer profitability is a crucial element for the success of CRM (4). In recent years, there has been a growing interest in application of CRM in healthcare industry by administrators. European public healthcare systems are implementing market-driven reforms such as managerial decentralization, the separation of purchasers and providers, the use of prospective payment systems, mostly performance-based, and the implementation of policies that allow patients to choose their hospital to improve the efficiency and quality of their services (5,6). These cause some kind of competition among hospitals. It is expected that patients will choose the hospitals with the best quality or, at least, will try to avoid ‘bad’ hospitals, and that ‘good’ hospitals will be rewarded with more resources (7). In the healthcare sphere, the difficulties of measuring health outcomes makes the evaluation of efficiency a controversial topic so health outcomes are usually replaced by output data (8). Patient satisfaction has become an important perspective in the measurement of health service outcome (9). 

Understanding more about high-value and potential patients, including the hospital departments and services that they utilize, will help hospitals appropriately design resource allocation strategies for health care services (2). For discovering all groups of patients, customer lifetime value (CLV) is an effective method that defines customers based on their characteristic, preference and activities. CLV referred to the profitability attributed to a customer during the time that the customer will stay with a company. CLV is applied in the literature as segmentation tools of customers in different industries (10, 11, 12, 13, 14, and 15). In this research, RFM technique is used to calculate CLV of the customers to segment them in a public sector hospital in Tehran. The main goal of the segmentation is who are the target customers and whose contributions are more significant in the patients of the hospital. In this study, adopted RFM analysis named RFML (Recency, Frequency, Monetary and Length) prescribes a segmentation of patients in the hospital by using a clustering–classification model. We used Two-step method to predict optimum K number of clusters, then K-means algorithm to cluster the patients. Also two approaches are examined for classification. In the first the output of clustering (cluster number of each patient) is used as decision attribute for Decision tree algorithm. And second, LTV value calculated by weighted RFML model categorized all customers into K categories. These categories are considered as decision attribute for Decision tree algorithm. We believe, greater LTV value shows more loyal patient. Two-step and K-means algorithms are used to identify all segments of patients, and decision tree algorithm to detect hidden knowledge rules and health care shopping behaviors by characteristics and behavioral information of patients. This result helps to examine the best marketing strategies for health care services. Proposed model are examined on an empirical study in a big public sector hospital in Iran. 768,864 data records are extracted of Hospital Information System (HIS) from 2009 to 2012. The main idea of this paper is, there is contrast between patient and customer loyalty. Three purposes involved to decrease that: 1) only, outpatient data are used 2) long time period is considered to monitor patients’ behavior (four years, from 2009 to 2012) that causes, 3) too many data records of patients (768,864 data records of 234,690 patients) for evaluation.

*Background*

*Customer relationship management (CRM)*

CRM is a philosophy that anticipates customer needs with the purpose of providing the target customers with the right product, at the right time, in the right place (16). Enterprises that succeed in correctly assessing customer value can offer customized services to diverse customers, perform effective customer relationship management and, simultaneously, also increase enterprise revenues (17). Under the concept of CRM, customers are not equal and, thus, it is unreasonable for the company to provide the same incentive offers to all customers. Instead, companies can select only those customers who meet certain profitability criteria based on their individual needs or purchasing behaviors (18). CRM framework can be classified into operational and analytical (17, 19, 20). Operational CRM refers to the automation of business processes, whereas analytical CRM refers to the analysis of customer characteristics and behaviors so as to support the organization’s customer management strategies. As such, analytical CRM could help an organization to better discriminate and more effectively allocate resources to the most profitable group of customers (21).

*CLV, RFM , wRFM and eRFM definitions*

Customer lifetime value (CLV) is typically used to identify profitable customers and to develop strategies to target customers (22). Generally, RFM (Recency, Frequency, and Monetary) method has been used to measure CLV or loyalty (23-25). Hughes proposed RFM analytic model in 1994 (26). Three variables of the model are (27):

1) Recency of the last purchase (R): refers to the interval between the time that the latest consuming behavior happens and present. The shorter the interval is, the bigger R is.

2) Frequency of the purchases (F): refers to the number of transactions in a particular period, for example, two times of one year, two times of one quarter or two times of one month. The many the frequency is, the bigger F is.

3) Monetary value of the purchases (M): refers to consumption money amount in a particular period. The much the monetary is, the bigger M is.

Wu & Lin (28) in their research showed the bigger value of R and F is, the more likely the corresponding customers are to produce a new trade with enterprises. Moreover, the bigger M is, the more likely the corresponding customers are to buy products or services with enterprises again. Liu and Shih (25) proposed a weighted RFM based method (wRFM) that is 

Cj= W_R_R_cj_+ W_F_F_cj+ _W_M_M_cj_, where W_R_, W_F_, W_M_ are the relative importance of the RFM variables. wRFM integrates analytic hierarchy process method (AHP) and data mining to recommend products based on customer lifetime value. The wRFM based method employs association rule mining to identify recommendation rules from customer groups that are clustered according to weighted RFM values. There are two opposing views about weights of the three variables of RFM in the literature that they are identical or not. But some research indicated that recency, frequency and monetary are different in the importance due to the characteristic of the industry (29, 30). eRFM refers to adopting or extended model of RFM depends on the industries. Decision makers can effectively identify valuable customers and then develop effective marketing strategy by adopting RFM model (27). Recent studies suggest that the predictability of RFM models can be improved via adding additional variables when predicting customer behaviors (31, 32). Table 1 shows the recent researches of customer segmentation with RFM method and data mining techniques.

*Data mining tools*

Data mining tools are a popular means of analyzing customer data within the analytical CRM framework. Many organizations have collected and stored a wealth of data of their customers. However, the inability to discover valuable information hidden in the data prevents the organizations from transforming these data into valuable and useful knowledge (33). 

**Table1:** The recent researches of RFM method and data mining techniques in different industries

**RFM/eRFM/** **wRFM**	**goal**	**Research field**	**Data mining technique**	**literatures**
WRFM	product recommendations to each customer group	Marketing in a business	Clustering(k-means) and association rules	Duen-Ren Liu and et al.(2005)
RFM	find out the characteristic of customer in order to strengthen CRM	electronic industry	Clustering (K-means) and classification(rough set theory-LEM2)	Ching-Hsue Cheng and et al. (2009)
RFM	Sequential pattern mining to discover customers’ purchasing patterns over time.	on-line retailers of electronic commerce	Sequential Pattern mining	Yen-Liang Chen and et al. (2010)
RFM_ WRFM	Cluster analysis to assess the customer loyalty	SAPCO Company	Clustering (K-means)	M. Seyed Hosseini and et al.(2010)
RFM_CRFM (C: count )	Estimating customer lifetime value	a health and beauty company	Clustering (K-means)	M. Khajvand and et al. (2011)
GRFM (G: group refers to the customers’ purchase patterns as well as the characteristics of products)	discover better customer consumption behavior and building a personalized purchasing management system	computer products	Clustering(PICC- Purchased items Constrained Clustering)	Hui-Chu Chang and et al.(2011)
RFM_RFMDR (D: discount and price, R: return times)	mine association rules of customer values	Online shopping	association rules (Supervised Apriori algorithm)	Wen-Yu Chiang and et al.(2011)
RFM	Identifying patients in target customer segments	hospital	Clustering (K-means) and classification(rough set theory)	You-Shyang Chen and et al.(2012)
LRFM (L: length, refers to the first and the last visit dates)	market segmentation of a children’s dental clinic	dental clinic	Clustering((SOM) technique)	Jo-Ting Wei and et al.(2012)
WRFM	discover sequential patterns	applying the synthetic data generation algorithm	PrefixSpan algorithm (the conventional sequential pattern mining method)	Ya-Han Hu (2012)

Data mining tools could help these organizations to discover the hidden knowledge in the enormous amount of data (21). In general, data mining has two major functions: the first one is to predict future tendency from an established model; the other is to locate unknown models from data. Models include: association, classification, estimation, prediction, affinity grouping, clustering, description and profiling (34). Lee (35) used Decision Tree and Association Rule tools to integrate the concepts of sequential pattern mining to extract features of Asthma attacks and predict chronic disease attacks. Abdelfattah (36) with data mining and serum examination and radioactive treatment, predicted whether Type C pneumonia pathologically changed to cirrhosis. Huang and Chen (37) used various data mining technologies to help machines learn to distinguish types of glaucoma. Lin (34) with Two-stage cluster analysis (Ward’s method and K-means) and decision tree analysis on 501 abnormal diagnoses in an emergency department found that nursing personnel make more frequent triage diagnoses than physicians do. The study of Yeh (38) analyzed dialysis patients' biochemical data to develop a decision support system to predict hospitalization of Hemodialysis patients and to suggest immediate treatments to avoid hospitalization. The research of Yang (39) used classification and decision tree to analyze the prediction model of patients’ demand in the Emergency department from real treatment situations. Also, many studies used data mining techniques to segment customers to identify potential and loyal customer in all industries. In the follow we explore some data mining models that used in the paper.

*Classification*

*Decision tree application*

Classification is one of the most common learning models in data mining (40, 41, 42). It aims at building a model to predict future customer behaviors through classifying database records into a number of predefined classes based on certain criteria (40, 17, 43, 44). Decision tree is an important technique is used extensively in classification. The advantages of decision tree theory include:

1) Can produce understandable rules;

2) Perform tasks without much computing

3) Can handle continuous and categorical variables

4) Can learn which attributes are important for classification (43).

Decision tree algorithm has been used in several studies for the purpose of customer database segmentation (45, 46). A CHAID decision tree subsequently splits customers into subgroups based on the chi-square statistic, identifying which variable splits the data best and whether further splitting induces a statistically significant improvement (47). Input of the classification algorithms is a table of objects with attributes which contain values for each object. One attribute is chosen to be the decision attribute, and then the remaining attributes are the condition attributes. This research calculated decision attribute with two approaches. Section 3 will explain this in detail.

*Clustering: K-means application*

Clustering is the process of grouping a set of physical or abstract objects into groups of similar objects (27). A cluster is a collection of data objects that are similar to one another within the same cluster and are dissimilar to the objects in other clusters (48). It is different to classification in that clusters are unknown at the time the algorithm starts. K-Means is one of the well-known algorithms for clustering which is very sensitive to the choice of a starting point for partitioning the items into K initial clusters (31).

*Research framework*

*constraints in the health care industry*

A patient is considered as a customer in health care industry in the literatures. This means a health care organization is equal to a business. So health care services marketing is an important concern of decision makers and stockholders. However there are some differences between patient going to a hospital and a customer going to a shop for buying goods or services. The major differences are:

1- Customer needs considered as permanent needs such as food and other things that need for living and comfort. But patient needs considered as case based temporary needs that may occur suddenly.

2- There is negative value of customer life time value in health care industry. Because patients preferred to improve as soon as possible. In other words short customer life is preferable. This is in contrast with traditional customer life time value.

3- Most of time patients are referred to the special hospital for various reasons such as: doctor prescription or order, lack of adequate equipment for specific disease or hospitalization and emergency department or triage referring and etc. 

These differences are obstacles to behave patient as customer in marketing strategies in health care services but many researchers don’t assist them. This paper tried to minimum this conflict, therefore considers three conditions for analyzing patients information. First, only outpatient data (from emergency departments and medical clinics) are extracted. Second, a longer period is considered for extracted data from HIS (from 2009 to 2012) that finally, causes the large data records of patients for evaluating (768,864). In these circumstances we can see to a patient as a customer but even may be the cases that didn’t satisfy the conditions which the long period time and large data decrease this contrast.

**Table 2:** Descriptive statistics of the model variables

**Field**	**Min**	**Max**	**Mean**	**Std. Dev**	**Variance**	**Median**	**Mode**	**Valid**
**Recency**	1	1511	924.806	394.597	155706.876	972	1506	234674
**Frequency**	1	50	2.612	3.933	15.468	1	1	234674
**Monetary**	1000	62272600	440167.1	1776964.302	3.1576E+12	72400	34000	234674
**Length**	0	1507	136.054	297.359	88422.609	0	0	234674

*Empirical study*

Shohadaye Tajrish Educational Hospital (ST Hospital) founded in 1966 on Tehran, Iran has more than twenty specialist clinics and four hundred beds considered as a big public sector hospital. Data records are extracted from hospital information system (HIS). Due to the nature of treatment industry and contrast between customer loyalty and patient loyalty, we select outpatient data records (emergency departments and medical clinics) to analyze patients’ behavior to detect all kind of the hospital customers and level of the loyalty. The time period taken in this study is four years. Period of time has long been considered as possible to increase transparency of patient behavior. The number of total outpatient transactions is 768,864 from September 2008 to October 2012. The study collects the data set of 234,690 patients who visited these emergency clinics in four years period.

*Weighted RFML*

The RFM model has been applied to various industries but it is rarely used in the context of the health care industry (2). Some researches try to develop RFM model and add some parameters to these three parameters (49) to extend the model for adapting to the industry. This study adopts L variable (length: time between first and last patient hospital admission) to custom RFM concept for hospitals. Added attribute make the balance among other attributes in the patient behaviors. For example, low frequency in a long time period didn’t show lower loyalty in the health care services, so length attribute compensate this. The descriptive statistics for R, F, M and L are presented in Table 2. If the first and the last visit dates are identical, the length is coded as 0. For the recency, the larger recency value is, the more recent the patient visits. Analytic hierarchy process (AHP) was used to determine the relative importance or weights of the RFML variables, W_R_, W_F_, W_M_ and W_L._ We used questioner and interview to determine efficient factors on patient loyalty that add to RFM model and weights of the extended model from administrative managers and medical directors of ST Hospital (Churn and Expected value are two proposed variables that eliminated and Length variable added). According to the assessments, the relative weights of the RFML variables are 4.5, 8.3, 8.2 and 4.9 respectively (scale is 1-10). 

*Structure of the model*

**Figure 1:**
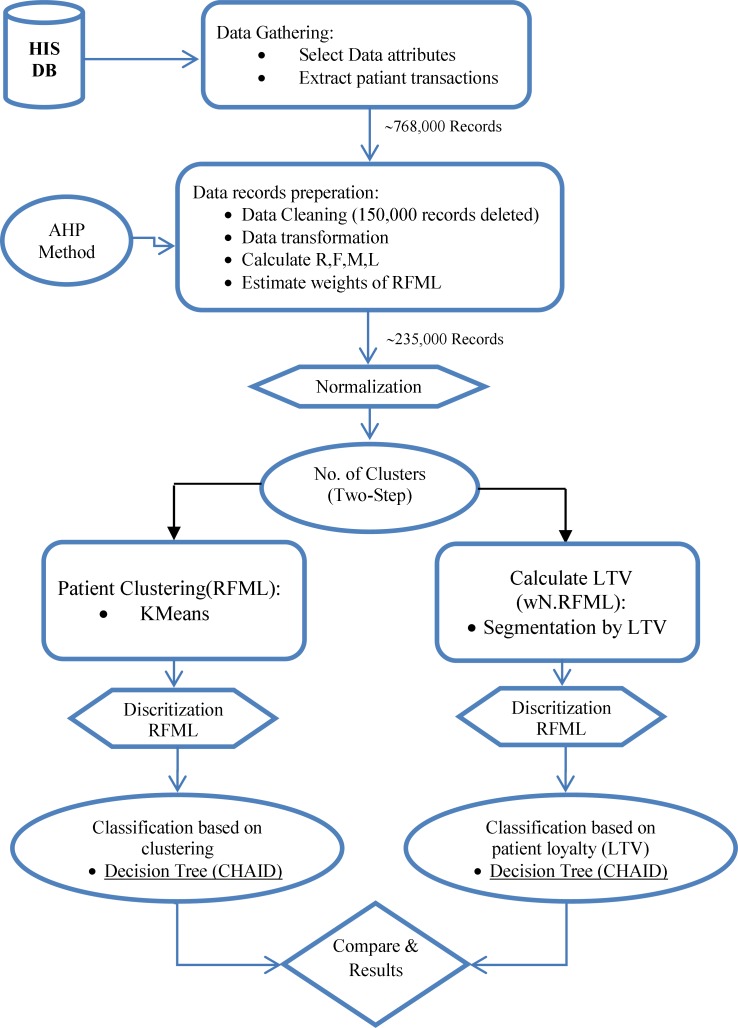
Flowchart of the proposed classification model

Structure of the model is shown as flowchart in Figure 1.

Step 1. Data records collection (initial data): The study selected data fields or attributes based on the RFML model from outpatient datasets of the HIS. These required features of the patients include: ID, age, gender, date of referring or admission, type of used services and the amount paid. In the first, datasets of ST Hospital are preprocessed to extract the customer transactions. 768,864 data records that belonged to 234,690 patients are collected. Each record contains information about each patient admission or transaction.

Step 2. Data records preparation (ready data): Data preprocessing is an inevitable step in knowledge discovery process. This step first, eliminates records which include noisy, inaccurate and missing values. In this case more than 150,000 records are deleted. These records include incorrect values such patient referrals with zero payment or invalid date and etc. Second, some features of data records are transformed into a format to be ready for next step. For example date of admission transformed to the number. Finally, RFML variables are calculated based on all transactions of each patient that make a table with 234,690 records. Also according to the assessments obtained by the AHP, the relative weights of the RFML variables are determined.

Step 4. Data normalization.

We normalize R, F, M and L variables to be consistent in terms of numerical unit. So they are standardized to the interval [0, 1]. The equations for normalization are shown below (2): 

V= V_i_ / Max (V_i_) that V={R, F, M, L}

Step 5. Determination of the cluster number (K)

Despite the classification algorithms, the segments are unknown in the clustering algorithm. But the number of segments must be defined by user in some algorithms such K-means. In this study Two step method is used to predict the optimum K or number of clusters. Two step algorithm don’t need the exact number of clusters which was defined by user while it finds the optimum number of cluster in a range which defined by user (49). Here, the proposed optimum cluster number is 5.

Step 5-1. Patient Clustering

The general purpose of clustering attempts to maximize the inter-cluster distance (to ensure the clusters are well separated) while minimizing the intra-cluster distance (to ensure compactness of the clusters) (2).

According to quantitative value of normalized R, F, M and L attributes for each patient, data partitioned into 5 clusters using the K-means algorithm (based on two ways: non weighted and weighted parameters). Table 3 shows the result of this clustering (for better understanding, principle value of variables are shown). Also importance of RFML variables in 5 clusters is shown in figure 2. In addition to find all segments of the customers and identify profitable patients, there is main reason for performing this step. The output of running K-means algorithm is a column which identifies each patient is belonged to which segment. This is the value that will be used as decision attribute for classification algorithm in the step 5-1-2.

**Table 3:** Descriptive statistics of five clusters

				**Frequency**		**Length**		**Monetary**		**Recency**	
		Total N	%	Mean	Standard Deviation	Mean	Standard Deviation	Mean	Standard Deviation	Mean	Standard Deviation
**Kmeans**	**cluster-1**	96609	41.17	1.58	1.43	21.08	66.82	225826.7	1190472	523.2	220.83
	**cluster-2**	3396	1.45	25.84	8.66	1134.49	254.3	3957069	5407150	1407.77	118.36
	**cluster-3**	99675	42.47	1.67	1.47	18.47	49.11	377182.2	1639632	1191.51	187.53
	**cluster-4**	14060	5.99	6.87	3.91	987.44	188.3	842459.5	1780686	1345.89	133.05
	**cluster-5**	20934	8.92	5.23	3.95	492.73	149.13	888509.2	2576546	1147.11	222.46
	**Total**	234674	100	2.61	3.93	136.05	297.36	440167.1	1776964	924.81	394.6

**Figure 2:**
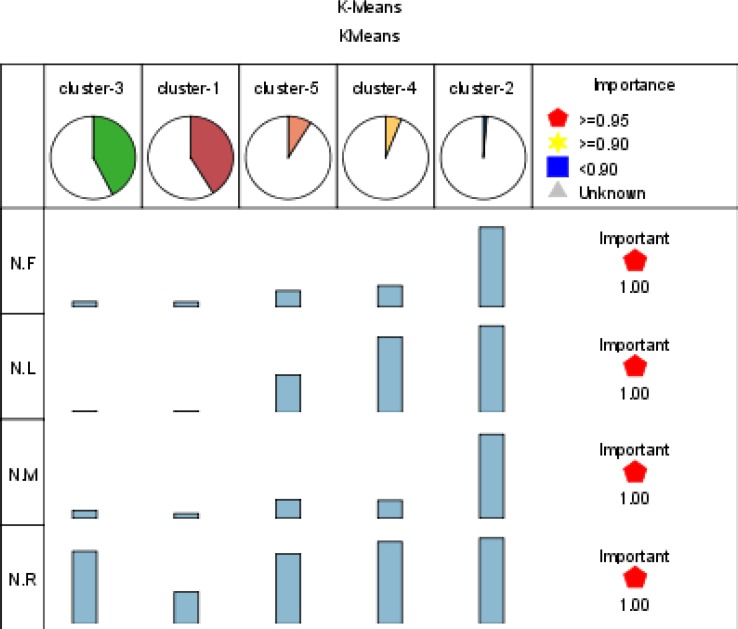
Importance of RFML variables in 5 clusters

Step 5-2. Estimation of CLV

The proposed optimum K is used to categorize all patients by customer value or CLV to K segments. The output of this segmentation will be used as decision field for classification. In the first, CLV is calculated based on weighted RFML method for each customer by equation 3. Relative importance of the variables is 4.5, 8.3, 8.2 and 4.9 respectively. 

CLV_Ci_= NR_ci_*W_R _+ NF_ci_*W_F_+ NM_ci_*W_M_+ NL_ci_*W_L_ (equation 3)

**Table 4:** Discrete values of continuous attributes in RFML model

variables	Range	Discrete value
Recency_TILE5	[1-543), [543-826), [826-1097), [1097-1323), [1323-1511]	R1, R2, R3, R4, R5
Frequency_TILE5	[1-2), [2-3), [3-4), [4-5), [5-50]	F1, F2, F3, F4, F5
Monetary_TILE5	[1000-34100), [34100-51002), [51002-111101), [111101-410300), [410300-62272600]	M1, M2, M3, M4, M5
Length_TILE5	[0-1), [1-2), [2-3), [3-167), [167-1507]	L1, L2, L3, L4, L5

Second, patients are categorized into 5 segments (K optimum number of Two step algorithm) based on CLV of each patient. Now we have loyalty attribute for each patient that show customer class based on CLV value. Finally, this attribute is considered as decision field for classification in the step 5-2-2.

Step5-1-1 & Step 5-2-1. Data discretization 

For improving the classification accuracy and solving the challenge of generating a large number of decision rules in the segmentation models, attributes are granulated (2). Applying discritization to data decrease further processing time of many method as well as increase quality of results (50). Continuous attributes in pervious step are granulated using Binning method. The Binning discretization based on the tiles method (equal count) is implemented to discretize all continuous attributes. Binning method can be useful for create categorical inputs to increase performance of classification method and data privacy. Table 4 shows discrete values of continuous attributes in RFML model.

Step5-1-2 classification by clusters (approach 1) 

Decision tree algorithm (CHAID) is used to generate the decision rules set from the experimental dataset for classifying ST hospital patients. Practically, a classification model is first built on a training set. Afterwards, the model is validated using previously unseen data, the test set (51). The experimental dataset is randomly divided into two groups, data serving as a training set and the remaining serving as a testing set. The generated rules sets supports the ‘if-then’ rules set as the knowledge-based system to determine the decision- making strategy and intelligently offer explanatory power (2). In the first, all classification algorithms needed condition attributes and decision attribute. These attributes are chosen based on health care experimental knowledge and expert point of view. In this case, decision field is cluster number of patient that determined in the step 5-1. And discretized recency, frequency, monetary and length are condition fields for running CHAID method. Efficacy of the input variables is shown in Figure 2. 

**Figure 2.**
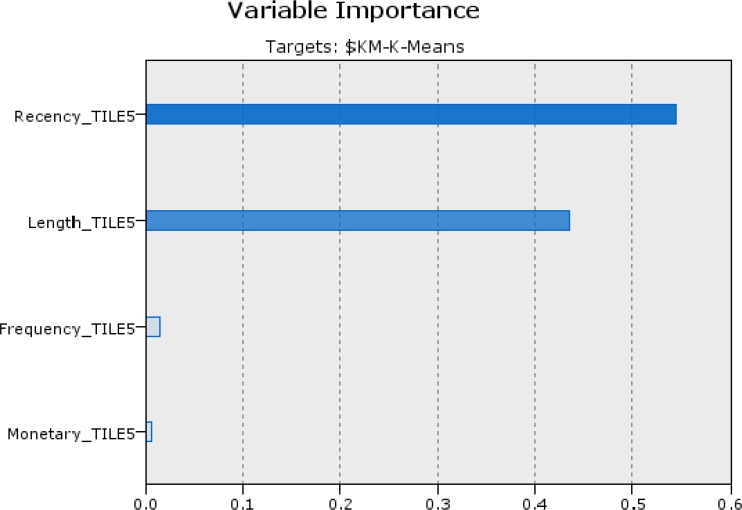
Importance degree of the variables in ascending order is: R=0.5449, L=0.4345, M=0.0148 and F=0.0058 in the classification by clusters method

Step 5-2-2 classification by CLV (approach 2)

**Figure 3:**
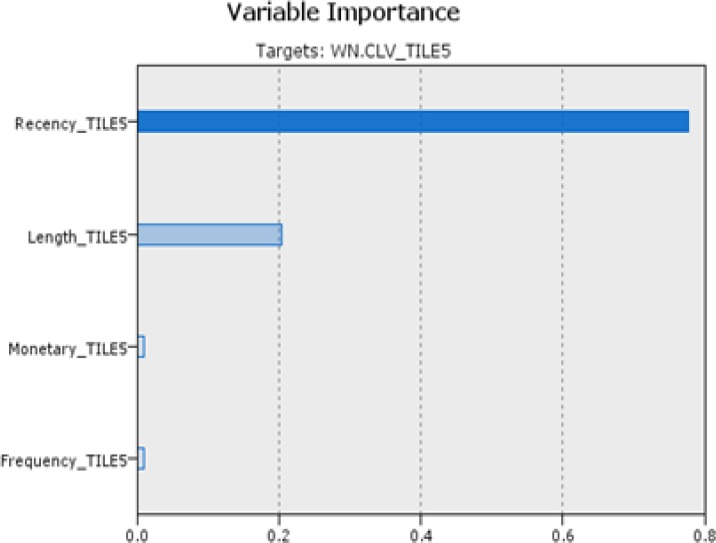
Efficacy of the variables in ascending order is: R=0.7766, L=0.2046, M=0.0096 and F=0.0092 in the classification by CLV

In the approach, customers are classified with CHAID algorithm based on CLV categories as decision field (step 5-2). Figure 3 shows the degree of impact and importance of the variables in the classification by CLV method. In the following we compare the result of two approaches of classification.

Step 8. Compare & extract rules

Order of variables importance in the both methods is the same. This shows recency and length respectively have the greatest impact on identify target customers in health care services. Results of output fields of two approaches by comparing actual and predicted class (coincidence matrix and performance evaluation) displayed the first approach, classification by k-means, has more accuracy (89.69%) than classification by CLV (80.98%). This experiment was examined by C5 and neural network algorithms for evaluations. Performance evaluations results are shown in table 5. As can be seen, Decision tree by CHAID algorithm performed better other methods in accuracy. In the following, outcomes of extracted rules of selected approach were analyzed.

**Table 5:** Comparison of the models accuracy and other algorithms

method	Accuracy (%)
Classification by clustering (decision tree - CHAID)	89.69%
Classification by CLV(decision tree- CHAID)	80.98%
Decision tree-C5	89.58%
Neural network	89.31%

*Findings*

*clustering analysis*

Table 3 shows descriptive statistics of 5 clusters including, number of patients, percentages, average and standard deviation of all parameters (R, F, M and L). The results indicate meaningful relations among attributes in the clusters. Cluster 2 has the best customers with high value in all attributes. These profitable customers are 1.45% of all patients (234, 674) who indicate loyal patients of the hospital. This is a fact that greater contributions and revenue can be achieved by fewer target customers (according Paretto rule). Length and recency factors indicate these patients were active during the total period that considered in this study (four years) and also have maximum number of usage (in average 25). Cluster 1 has the customers with lowest value of all attributes and considerable numbers (41.17%). These patients have one time referrals in average and more than three years have been inactive that means customer churn. Other clusters (3, 4 and 5) show the patients that may be return again and have readmission (because of recently admission), so the hospital managers must be establish better relationship to improve satisfaction of these customers. 

**Table 6:** Extracted rules from classification model based on k-means clustering

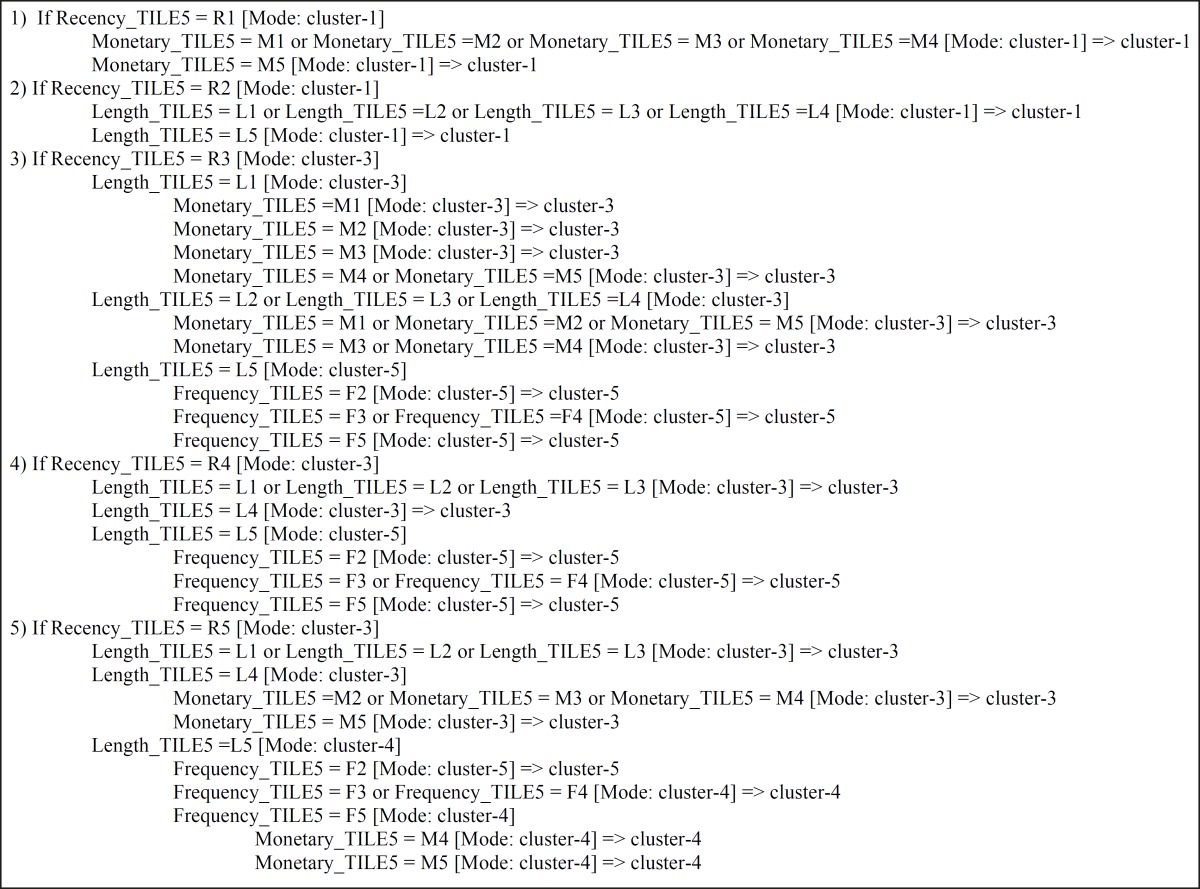

*Extract and interpret the rules*

CHAID method based on importance degree of input variables creates a decision tree. Decision tree in this experiment has depth of 4 that Recency_TILE5, Length_TILE5, Frequency_TILE5 and Monetary_TILE5 variables are entered respectively (with 0.54, 0.43, 0.01 and 0.01 importance). Table 6 shows extracted rules from ST hospital dataset by CHAID algorithm based on k-means clustering. As follows from this table there are meaningful relations among recent lookup of patient (recency), time duration that patient refer to the hospital for services (length), the number of times for visiting (frequency) and total fees paid by patient (monetary). The analytic results of treated patients within four years on ST hospital show interesting knowledge as described below. It should be noted the time period considered in the study is 4 years.

- All patients that last visit were about 947 days ago (about 3 years ago- first year of the considered period time of this study) definitely fall in cluster 1 or churn customers. The number of churn customers are considerable (41.17%). But the hospital loses them and must spend a lot of money for acquisition of this market segment. Hospital management should try to compensate this by improving quality of the services and strong customer relationship to cause less churn rate from now.
- Patients that last visit were about 1.5 years ago (second year of the considered period time of this study) with low and medium monetary (<M5), despite the low value of frequency and length attributes can be valuable customers as low value patients. The number of this segment is considerable (42.47%). Due to temporary needs of people to health care services this market segment may return and use the hospital services when they need. Due to the large number of this patients and likelihood of readmission, the administrators can guarantee significant profitability in the future by spending money to build a strong relationship with these customers.
- Patients that last visit were about 6-7 month ago with long time activation (L5), upward monetary (>=M4) and minimum number of visit 4 times (F>=F4) are very high value customers. This segment is profitable following of loyal customers but the number is more than loyal patients (6% of all customers). The hospital management should operate on these patients has become loyal customers by introduce new, free and supplementary services.
- Patients that last visit were about 1 year ago with long term activation (L5) and frequency more than 2 times (>=F3) are high value customers. The result shows monetary of this patient are significant.
- Ranking of all segment based on meaning rules extracted from decision algorithm (CHAID) are: loyal segment, very high value, high value, medium value and churn group in ascending order that the number of each segment are: 1.45%, 5.99%, 8.92%, 42.47% and 41.17% respectively. The administrators should adopt appropriate strategies to suit each group in order to further profit for the hospital.

**conclusion and suggestion for marketing development**

Nowadays, health care industry like other industries should interact with patients as customers and implement strong marketing strategies for equation and retention of them. There are some differences between patient and customer needs and requirements which must be considered for assessment them. This research develops a classification method based on weighted eRFM (RFML) with clustering and customer life time value (CLV) as two approaches. The results show classification based on clustering (k-means) has more accuracy than CLV method. The outcomes of the study identify all patients’ classes of ST hospital and behavior of them. More revenue of the hospital generated from less than 1.5% all customers as loyal patients, so the managers should adopt special and custom relations to hold and retention of the profitable market. A large number of patients placed in churn group (41.17%) that shows such shortcoming or low quality of hospital services. Also the result shows there is a significant group (42.47%) that recently visited with medium monetary and low frequency and length attributes who is a new opportunity for the hospital. The hospital administrators should establish excellent relationship and implement marketing strategies to improve satisfaction of this new market. This article highlighted the role of Customer Relationship Management in the hospitals and proposed a classification model for identifying all kind of patients to improve marketing strategies. Further research will focus on the effective factors and reasons on choosing hospital by patients with aim of services quality improvement. 

## References

[1] Kim SY, Suh EH, Hwang HS (2003). A model for evaluating the effectiveness of CRM using the balanced scorecard. J. Interact. Mark.

[2] Chen YS, HsueCheng C, JungLai C, YiHsu C, JhouSyu H (2012). Identifying patients in target customer segments using a two-stage clustering-classification approach: A hospital-based assessment. Comput. Biol. Med.

[3] Joo YG, Sohn SY (2008). Structural equation model for effective CRM of digital content industry. Expert. Syst. Appl.

[4] Lee JH, Park SC (2005). Intelligent profitable customers’ segmentation system based on business intelligence tools. Expert. Syst. Appl.

[5] Allen P (2009). Payment by results’ in the English NHS: the continuing challenges. Public. Money Manage.

[6] Botten G, Grepperud S, Nerland SM (2004). Trading patients lessons from Scandinavia. Health Policy.

[7] Garcia-Lacalle J, Martin M (2010). Rural vs urban hospital performance in a ‘competitive’ public health service. Soc. Sci. Med.

[8] Linna M, Hakkinen U, Magnussen J (2006). Comparing hospital cost efficiency between Norway and Finland. Health Policy.

[9] Seyedhamzeh S, Barkhi Darian B, Safarian M, Mousavi N, Norouzy A (2011). Predictors of patient satisfaction about hospital meals in Mashhad(IRAN). CLIN. Nutr. Suppl.

[10] Rosset S, Neumann E, Eick U, Vatnik N, Idan Y (2002). Customer Lifetime Value Modelling and Its Use for Customer Retention Planning. Proceedings of the eighth ACM SIGKDD international conference on Knowledge discovery and data mining..

[11] Hwang H, Jung T, Suh E (2004). An LTV model and customer segmentation based on customer value: a case study on the wireless telecommunication industry. Expert. Syst. Appl.

[12] Shin HW, Sohn SY (2004). Segmentation of stock trading customers according to potential value. Expert. Syst. Appl.

[13] Kim SY, Jung TS, Suh EH, Hwang HS (2006). Customer segmentation and strategy development based on customer life time value: a case study. Expert. Syst. Appl.

[14] Haenlien M, Kaplan AM, Beeser AJ (2007). A Model to Determine Customer Lifetime Value in a Retail Banking Context. E. M. J.

[15] Benoit Dries F, Van den PD (2009). Benefits of quantile regression for the analysis of customer lifetime value in a contractual setting: An application in financial services. Expert. Syst. Appl.

[16] Yourdon E (2000). CRM: An introduction. CUTTER. IT. J.

[17] Berson A, Smith S, Thearling K (2000). Building data mining applications for CRM.

[18] Dyche J, Tech J (2001). The CRM Handbook: A Business Guide to Customer Relationship Management.

[19] He Z, Xu X, Huang JZ, Deng S (2004). Mining class outliers: Concepts, algorithms and applications in CRM. Expert. Syst. Appl.

[20] Teo TSH, Devadoss P, Pan SL (2006). Towards a holistic perspective of customer relationship management implementation: A case study of the housing and development board, Singapore. Decis. Support. Syst.

[21] Ngai EWT, Xiu L, Chau DCK (2009). Application of data mining techniques in customer relationship management: A literature review and classification. Expert. Syst. Appl.

[22] Irvin S (1994). Using lifetime value analysis for selecting new customers. Credit World.

[23] Miglautsch J (2000). Thoughts on RFM scoring. J. Database. Market.

[24] Kahan H (1998). Using database marketing techniques to enhance your one-to-one marketing initiatives. J. Consum. Mark.

[25] Liu DR, Shih YY (2005). Integrating AHP and data mining for product recommendation based on customer lifetime value. Inform. Manage.

[26] Hughes AM (1994). Strategic database marketing.

[27] Cheng CH, Chen YS (2009). Classifying the segmentation of customer value via RFM model and RS theory. Expert. Syst. Appl.

[28] Wu J, Lin Z (2005). Research on customer segmentation model by clustering. ACM International Conference Proceeding Series.

[29] Tsai CY, Chiu CC (2004). A purchase-based market segmentation methodology. Expert. Syst. Appl.

[30] Stone B (1995). Successful direct marketing methods.

[31] Seyed Hosseini SM, Maleki M, Gholamian MR (2010). Cluster analysis using data mining approach to develop CRM methodology to assess the customer loyalty. Expert. Syst. Appl.

[32] Yeh IC, Yang KJ, Ting TM (2008). Knowledge discovery on RFM model using Bernoulli sequence. Expert. Syst. Appl.

[33] Berson A, Smith S, Thearling K (2000). Building data mining applications for CRM.

[34] Lin WT, Wang ST, Chiang TC, Shi YX, Chen WY, Chen HM (2010). Abnormal diagnosis of Emergency Department triage explored with data mining technology: An Emergency Department at a Medical Center in Taiwan taken as an example. Expert. Syst. Appl.

[35] Lee CH, Yu Chen JC, Tseng VS (2011). A novel data mining mechanism considering bio-signal and environmental data with applications on asthma monitoring. Comput. Meth. Prog. Bio.

[36] Abdelfattah MA, El-Shahat AT, Ahmad ME, Mosaad AA, Mohamed MO, Gamal ES (2006). Discriminant function based on hyaluronic acid and its degrading enzymes and degradation products for differentiating cirrhotic from non-cirrhotic liver diseased patients in chronic HCV infection. Clin. Chim. ACTA.

[37] Huang ML, Chen HY (2005). Development and comparison of automated classifiers for glaucoma diagnosis using stratus optical coherence tomography. Invest. Ophth. Vis. Sci.

[38] Yeh JH, Wu TH, Tsao CW (2011). Using data mining techniques to predict hospitalization of hemodialysis patients, Decis. Support. Syst.

[39] Yang CC, Lin WT, Chen HM, Shi YH (2009). Improving scheduling of emergency physicians using data mining analysis. Expert. Syst. Appl.

[40] Ahmed SR (2004). Applications of data mining in retail business. Information Technology: Coding and Computing.

[41] Berry MJA, Linoff GS (2011). Data mining techniques: for marketing, sales, and customer relationship management.

[42] Carrier CG, Povel O (2003). Characterising data mining software. Intelligent Data Analysis.

[43] Chen YL, Hsu CL, Chou DC (2003). Constructing a multi-valued and multi labeled decision tree. Expert. Syst. Appl.

[44] Mitra S, Pal SK, Mitra P (2000). Data mining in soft computing framework: A survey. IEEE. T. Neural. Network.

[45] Galguera L, Luna D, Mendez MP (2006). Predictive segmentation in action - Using CHAID to segment loyalty card holders. Int. J. Market. Res.

[46] McCarty JA, Hastak M (2007). Segmentation approaches in data-mining: A comparison of RFM, CHAID, and logistic regression. J. Bus. Res.

[47] Coussement K, Van den Bossche FAM, De Bock KW (2014). Data accuracy's impact onsegmentation performance: Benchmarking RFM analysis, logistic regression, and decision trees. J. Bus. Res.

[48] Han J, Kamber M (2006). Data Mining: Concepts and Techniques.

[49] Khajvanda M, Tarokh MJ (2011). Estimating customer future value of different customer segments based on adapted RFM model in retail banking context. Procedia. Comput. Sci.

[50] Bazan JG, Nguyen HS, Nguyen SH, Synak P, Wr’oblewski J, Polkowski L., Tsumoto S, Lin T.Y (2000). Rough set algorithms in classification problem. Rough Set Methods and Applications: New Developments in Knowledge Discovery in Information Systems.

[51] Witten I, Frank E (2000). Data mining: Practical machine learning tools and techniques with java implementations.

